# Adjustments in purchasing arrangements to support the COVID-19 health sector response: evidence from eight middle-income countries

**DOI:** 10.1093/heapol/czad121

**Published:** 2024-02-09

**Authors:** Divya Parmar, Inke Mathauer, Danielle Bloom, Fahdi Dkhimi, Aaron Asibi Abuosi, Dorothee Chen, Adanna Chukwuma, Vergil de Claro, Radu Comsa, Albert Francis Domingo, Olena Doroshenko, Estelle Gong, Alona Goroshko, Edward Nketiah-Amponsah, Hratchia Lylozian, Miriam Nkangu, Obinna Onwujekwe, Obioma Obikeze, Anooj Pattnaik, Juan Carlos Rivillas, Janet Tapkigen, Ileana Vîlcu, Huihui Wang, Pura Angela Wee Co

**Affiliations:** Department for Population Health, School for Life Course and Population Sciences, King’s College London, Guy’s Campus, London SE1 1UL, UK; Department of Health Systems Governance and Financing, World Health Organization, Avenue Appia, Geneva 1211, Switzerland; Health, Nutrition, and Population Global Practice, World Bank Group, Washington D.C. 20433, USA; Department of Health Systems Governance and Financing, World Health Organization, Avenue Appia, Geneva 1211, Switzerland; Department of Public Administration and Health Services Management, University of Ghana Business School, Legon, Accra P.O. Box LG 78, Ghana; Health, Nutrition, and Population Global Practice, Europe and Central Asia Region, World Bank Group, 1818 H Street, N.W., Washington D.C. 20433, USA; Health, Nutrition, and Population Global Practice, Europe and Central Asia Region, World Bank Group, 1818 H Street, N.W., Washington D.C. 20433, USA; RTI International Philippines, 16F Strata 2000, Ortigas Center, Pasig City 1600, Philippines; Health, Nutrition, and Population Global Practice, Europe and Central Asia Region, World Bank Group, 1818 H Street, N.W., Washington D.C. 20433, USA; Philippine Health Insurance Corporation, 1704 Jose Laurel St, Manila 1005, Philippines; Health, Nutrition, and Population Global Practice, Europe and Central Asia Region, World Bank Group, 1818 H Street, N.W., Washington D.C. 20433, USA; Health, Nutrition, and Population Global Practice, Europe and Central Asia Region, World Bank Group, 1818 H Street, N.W., Washington D.C. 20433, USA; Health, Nutrition, and Population Global Practice, Europe and Central Asia Region, World Bank Group, 1818 H Street, N.W., Washington D.C. 20433, USA; Department of Economics, University of Ghana, University of Ghana Kumasi City Campus, Legon Accra P. O. Box LG57, Ghana; Health, Nutrition, and Population Global Practice, Europe and Central Asia Region, World Bank Group, 1818 H Street, N.W., Washington D.C. 20433, USA; Health Promotion Alliance Cameroon, Youandé, Cameroon; Health Policy Research Group, College of Medicine, University of Nigeria, Enugu Campus, Enugu 400001, Nigeria; Department of Community Medicine, Federal Medical Centre, No. 1 Hospital Road, Ovom, Yenagoa, Bayelsa 560231, Nigeria; ThinkWell, 1519 York Road, Lutherville, MD 21093, USA; Health, Nutrition, and Population Global Practice, Europe and Central Asia Region, World Bank Group, 1818 H Street, N.W., Washington D.C. 20433, USA; International Doctoral Programme in Epidemiology and Public Health, Tampere University, Kalevantie 4, Tampere 33100, Finland; ThinkWell, Regus, Nations Business Centre, 6th floor Rue du Pré-de-la-Bichette 1, Geneva 1202, Switzerland; Health, Nutrition, and Population Global Practice, Europe and Central Asia Region, World Bank Group, 1818 H Street, N.W., Washington D.C. 20433, USA; ThinkWell, Regus Plaza Bldg., United Nations Avenue, Manila 1000, Philippines

**Keywords:** COVID-19, health financing, health purchasing, middle-income countries

## Abstract

The COVID-19 pandemic has triggered several changes in countries’ health purchasing arrangements to accompany the adjustments in service delivery in order to meet the urgent and additional demands for COVID-19-related services. However, evidence on how these adjustments have played out in low- and middle-income countries is scarce. This paper provides a synthesis of a multi-country study of the adjustments in purchasing arrangements for the COVID-19 health sector response in eight middle-income countries (Armenia, Cameroon, Ghana, Kenya, Nigeria, Philippines, Romania and Ukraine). We use secondary data assembled by country teams, as well as applied thematic analysis to examine the adjustments made to funding arrangements, benefits packages, provider payments, contracting, information management systems and governance arrangements as well as related implementation challenges. Our findings show that all countries in the study adjusted their health purchasing arrangements to varying degrees. While the majority of countries expanded their benefit packages and several adjusted payment methods to provide selected COVID-19 services, only half could provide these services free of charge. Many countries also streamlined their processes for contracting and accrediting health providers, thereby reducing administrative hurdles. In conclusion, it was important for the countries to adjust their health purchasing arrangements so that they could adequately respond to the COVID-19 pandemic, but in some countries financing challenges resulted in issues with equity and access. However, it is uncertain whether these adjustments can and will be sustained over time, even where they have potential to contribute to making purchasing more strategic to improve efficiency, quality and equitable access in the long run.

Key messages

- All countries in the study adjusted their health purchasing arrangements, although to varying degrees. Yet, the lack of funding and implementation challenges limited the envisaged objectives of the adjustments in purchasing arrangements, with a major concern relating to inadequate benefits, increased out-of-pocket expenditure for COVID-19 services and the risk of reduced financial protection.
- Alignment across the different purchasing adjustments is crucial to ensure the provision of new health benefits and services as part of the pandemic response whilst maintaining the provision of essential health care.
- It is important to have pre-established governance arrangements that allow for accelerated decision-making on purchasing in emergencies. This should involve a broader set of stakeholders, such as health insurance funds, NGOs and civil society organizations.
- Purchasing arrangements need to have sufficient built-in adaptability and come along with an adequate information system to assess whether changes are meeting needs as a health emergency progresses and requirements evolve.

## Introduction

The COVID-19 pandemic has challenged the resilience of every country’s health system (Legido-Quigley *et al*., 2020; Haldane *et al*., 2021; Tessema *et al*., 2021; Mustafa *et al*., 2022). The provision of health services had to be reconfigured and adapted to meet the urgent and additional needs created by the pandemic. As cases surged, demand for COVID-19 services such as testing, contact tracing, intensive care and home care increased substantially (Goic *et al*., 2021; Litton *et al*., 2021). To accompany this reconfiguration of health service provision, countries had to adapt various aspects of health purchasing arrangements as part of the COVID-19 health sector response (Mathauer *et al*., 2022; Mathauer, 2023).

Purchasing in the health sector refers to the ‘allocation of pooled funds to health providers for the delivery of health services on behalf of certain groups or the entire population’ (Mathauer *et al*., 2019). Purchasing can be either passive or active (strategic) and is distinct from, but closely related to, procurement, the latter being the buying of medicines or medical supplies in bulk. Strategic purchasing implies that allocations of pooled funds to health providers are based, at least in part, on information related to provider performance and the health needs of the population they serve. There is growing consensus and evidence that purchasing health services must be more strategic in order to advance Universal Health Coverage (UHC) objectives, i.e. equitable distribution of resources, efficiency, transparency, equitable access to services, financial protection and quality of care (Hanson *et al*., 2019; Mathauer *et al*., 2019; Gatome-Munyua *et al*., 2022). For example, new insights are presented in a 2022 special issue on nine African countries’ path towards more strategic purchasing and the related challenges (Gatome-Munyua *et al*., 2022).

Several studies are available on the adjustments in purchasing in high-income countries. Notably, in European countries, a key policy response was to ensure that COVID-19 health services were free of charge (Thomson *et al*., 2022). Moreover, governments and public payers assumed most of the COVID-19-related financial risks of providers (Montás *et al*., 2022; Schmidt *et al*., 2022; Waitzberg *et al*., 2022), as in many countries hospitals received their usual budgets or additional funds to compensate for revenue shortfalls (Quentin *et al*., 2020). Overall, the evidence shows that the structure and financing of health systems affected the capacity of providers to cope with the pandemic (Waitzberg *et al*., 2021). However, there is scarce evidence on how purchasing arrangements and adjustments played out in low- and middle-income countries (LMICs) as part of their COVID-19 health sector response. Three overview papers indicate that most LMICs adjusted their purchasing arrangements and payment methods, and explored a wide range of different options; yet, in contrast to high income countries, they were often constrained in effectively offering an expanded benefit package free of charge due to financial shortages (Haldane *et al*., 2021; Gadsden *et al*., 2022; Mathauer *et al*., 2022). Governance and public finance, including payment of providers, are identified as key factors to support provision of COVID-19 health services (Haldane *et al*., 2021). Likewise, a compilation of case studies on sub-Saharan African countries found limited use of provider payments as a tool to advance national priorities and influence service provision during the pandemic (SPARC, 2022).

Building on this existing work, our paper presents a multi-country synthesis of adjustments made in purchasing arrangements in eight lower and upper middle-income countries (MICs) (Armenia, Cameroon, Ghana, Kenya, Nigeria, Philippines, Romania and Ukraine). These countries fall within the same broad income group but they have relatively more (financial and technical) means to respond to the COVID-19 pandemic as compared with low-income countries. Moreover, they all have a separate national purchasing agency, yet within an overall still fragmented health financing system.

Specifically, it examines changes in the key domains of purchasing arrangements namely, governance arrangements for purchasing, funding allocations, benefits design, provider payments, contracting and information management systems, and seeks to explore indications of the effects of these adjustments in relation to UHC objectives.

## Materials and methods

This paper is based on observational studies in eight lower and upper MICs (as per World Bank classification in 2021). We used the outputs produced in a Collectivity Project during 2021–2022 on ‘Strategic purchasing and COVID-19’, jointly facilitated by Results for Development (R4D), the World Health Organization (WHO) and the Strategic Purchasing Africa Resource Centre (SPARC). The Collectivity is a ‘community of practice’ platform that facilitates (virtual) collaborative projects to support collective learning based on joint interests and voluntary participation across LMICs. Members have diverse backgrounds and include academics from various disciplines, policymakers, health practitioners, staff from international organizations, international/country NGOs and independent consultants.

The ‘Strategic Purchasing and COVID-19’ Project was opened on The Collectivity website (www.thecollectivity.org) in February 2021 to solicit participation from Collectivity members to work in country teams focused on documenting strategic purchasing reforms that occurred in their countries during the pandemic. The selection of countries, therefore, was based on the country teams that signed up to work on this project. The country teams consisted of a mix of Collectivity members (practitioners and researchers), all with a unique and in-depth understanding of how their country’s health system managed purchasing decisions during the pandemic. The Ghana study was complemented by work undertaken by the WHO country office of Ghana applying deeper analysis using the same research questions. Key information about the country and the main purchasing agency is provided in a supplementary file (Supplementary Tables S1 and S2).

### Conceptual framework and research questions

The Collectivity country teams met virtually in February 2021 to discuss the project and review the proposed plan of collaboration. Several guiding frameworks related to strategic purchasing were shared and examined (Mathauer *et al*., 2019; Cashin and Gatome-Munyua, 2022; Strategic Purchasing Collectivity, 2022). These frameworks use very similar terms and conceptualizations, with benefits design (what to purchase), provider payments (how to pay) and contracting (from whom to buy) as the central domains of purchasing, with the accompanying information management systems for monitoring provider activity and resource allocation decisions as a decisive factor for making purchasing more strategic. Governance was also applied as an overarching health system function; it is particularly relevant for guiding strategic purchasing decisions and influences the other domains (World Health Organization, 2019).

This led the teams to co-develop the following overall research questions for the country studies:

1. What changes to purchasing arrangements were made as part of the COVID-19-health sector response and against what objectives?
2. What were the implementation challenges of these adjustments in purchasing arrangements?
3. Were there any indications that purchasing arrangements have positively affected UHC objectives, and what lessons can be drawn?

Based on this, a questionnaire was developed with more detailed questions to help ensure consistency across country studies.

### Data

We used secondary data, i.e. the outputs (summary country reports) produced by the Collectivity country teams. These are summary country reports, five out of these being published for Cameroon, Ghana (focusing on procurement), Philippines, Ukraine, Armenia and Romania (Wee-Co *et al*., 2021; Adin-Darko *et al*., 2022; Chukwuma *et al*., 2022; Doroshenko *et al*., 2022; Nkangu *et al*., 2022), a more detailed publication on Armenia and Romania (Chukwuma *et al*., 2023), whereas we used the non-published reports on Ghana (Abuosi and Nketiah-Amponsah, 2022), Kenya (Njoka *et al*., 2022) and Nigeria (Ayomoh *et al*., 2022). We triangulated this information during consultations with the country teams.

The basis for these country summary reports was a mix of qualitative and quantitative information, collected by each team (See Supplementary Table S3). The qualitative data included in-depth interviews with key informants based on purposive sampling. Key informants included representatives from the health ministries, purchasing agencies (such as the national health insurance schemes) and health workers. Documents reviewed included technical reports, policy documents, grey literature, research papers or press releases relevant to strategic purchasing in order to provide background knowledge of the prevailing country issues and the solutions proposed and implemented in response to the COVID-19 pandemic. The quantitative data were mostly secondary (e.g. budgets, claims data, utilization data). The teams collected data from March to November 2021. The period of policy responses considered thus ranged from the start of the pandemic to November 2021.

To ensure further alignment between approaches, timelines as well as the structure and focus of country summary reports across the country teams, they were supported by a Collectivity facilitator. Working with these facilitators, the teams developed their data collection approaches, which were shared with other teams for feedback. A series of virtual exchanges were then organized for teams to share interim findings, review challenges encountered during this work, discuss insights and lessons and ensure consistency for cross-country synthesis. Thereafter, country teams developed summary reports, with their findings organized along the key domains of purchasing (governance arrangements for purchasing, benefit design, provider payments and contracting and monitoring and information management systems). A final virtual consultation workshop was held on 14 December 2021 during which participants identified cross-cutting themes and lessons learnt.

### Data analysis

Thematic analysis of country reports was conducted using NVivo12. Documents were imported into NVivo12 and were coded according to key domains of strategic purchasing. For each domain, we examined the adjustments made as part of the COVID-19 health sector response, including the implementation challenges, and the effects of these adjustments in relation to UHC objectives. The sustainability and continuation of these adjustments in the future were also explored. Supplementary Table S4 (supplementary file) provides brief definitions of the domains and the thematic areas that guided the analysis. The analysis was an iterative process. While the initial coding was done by the first author in consultation with second author, thereafter, the country teams and Collectivity facilitators were involved in reflecting on any additional themes identified and in the interpretation and validation of findings.

## Results

Our evidence shows that the COVID-19 pandemic triggered changes in purchasing arrangements in all eight countries included in this project, whereby the emergency response to the pandemic involved adjustments in several purchasing domains. The country details presented in the results are taken from the country reports cited above.

### Modifications of governance arrangements

We found that in the early stages of the pandemic, all the countries in this study responded by modifying existing structures and/or establishing new governance structures to coordinate the overall national COVID-19 response. Most countries used centralized mechanisms for the COVID-19 response to enhance coordination within the health system, while in a few countries, governance structures at lower levels were also leveraged.

In all the countries, the broader national multi-sectoral response was directly led or backed by high-level political leaders or the executive department (Table 1). In two countries (Philippines, Kenya), these coordination bodies were chaired by the Ministry of Health. Membership in these committees primarily consisted of the public sector. In Nigeria, to ensure broad-based inclusivity, the Federal Ministry of Health, National Health Insurance Scheme, Nigeria Centre for Disease Control, State Ministries of Health and the National Primary Healthcare Development Agency played key roles as part of the response and coordination team with the national response coordinated by a multi-sectoral Presidential Task Force on COVID-19 response. In contrast, in Kenya and Ghana, the national health insurance schemes were not formally involved in decision-making processes.

**Table 1.** Governance arrangements for the national COVID-19 response

	Leadership for the COVID-19 response
Armenia	A newly formed Commandant Office, led by the Deputy Prime Minister, with representation across multiple ministries including the Ministry of Health (the leadership for the response was later delegated to the Ministry of Health)
Cameroon	Inter-Ministerial Committee on COVID-19
Ghana	Presidential Task Force on COVID-19
Kenya	National Emergency Response Committee (NERC), a newly established committee chaired by the Health Cabinet Secretary
Nigeria	Presidential Task Force on COVID-19 and Nigeria Centre for Disease Control
Philippines	Inter-Agency Task Force for the Management of Emerging Infectious Diseases (IATF-EID) chaired by the Department of Health (initially created to tackle the SARS epidemics)
Romania	The Ministry of Health established the Operational Coordination Center that communicated with public health directorates on essential information and needs related to COVID-19
Ukraine	Chief Sanitary Doctor, also a Deputy Minister of Health, was in-charge of the inter-ministerial Task Force on COVID-19

Some changes in governance arrangements impacted purchasing directly. For instance, in Armenia, an amendment to the Law on the Legal Regime of the State of Emergency authorized the Ministry of Health to assume oversight of all public hospitals as well as private facilities that were contracted to provide COVID-19 services. This allowed for central coordination of both COVID-19 and non-COVID-19 health services and thus increased accountability of these facilities vis-à-vis the State Health Agency (the government purchasing organization) and the Ministry of Health during the pandemic. In Romania, by a Presidential decree, the Ministry of Internal Affairs was authorized to conduct centralized procurement of personal protective equipment (PPE) and sanitation supplies to support health service purchasing.

In a few countries, existing governance structures at the sub-national level were used or revived to decentralize some purchasing decisions. For instance, in Cameroon, the Incident Management System was reactivated at the regional headquarters level, which was first operationalized during the 2018 cholera outbreak. District Medical Officers, who are incident managers at the district level, were allocated funds directly by the Ministry of Health to purchase medical supplies and health services—including local procurement of COVID-19 test kits and management of public treatment and prevention services. Regional delegations for public health had the responsibility to act as the main purchasers of COVID-19 services from the private sector. In Nigeria, the purchasing and provision of COVID-19 services in terms of testing, isolation and treatment were devolved to the state level and other institutions including tertiary health institutions like the Federal Medical Centers and teaching hospitals.

### Mobilizing and reallocating funds

A key concern in times of health emergencies is identifying sources of additional funding and ensuring that there is sufficient flexibility in their use. Countries used several tailored strategies to provide more funds to the health sector, ranging from the creation of special fund holding arrangements to manage COVID-19 resources, including extra-budgetary funds, activation of emergency funding or reallocation of funds from the overall government budget or from within the health sector budget. Governments also pursued using private sector and donor financing to provide additional resources for the pandemic response. Further, challenges over the sustainability of funding sources became apparent in many countries as the pandemic progressed.

In many countries in this study, purchasers (i.e. the Ministry of Health foremost) were allocated more funds via additional lending. In Romania, additional budget allocations were provided to the health sector via state resources and loans due to persistent budgetary pressure at the service delivery level. Likewise, in Kenya, the government raised substantial additional funding, mostly through an unprecedented level of external support including loans.

In others, changes allowed for special funding and fundholding arrangement to be adopted. For instance, Cameroon created a Special National Solidarity Fund to be shared across 24 ministerial departments. Moreover, existing general budget funds were also reallocated through a modification of the financial law to create more flexibility in use and encourage greater involvement of the formal private sector. In Armenia, declaring a state of emergency allowed the government to reallocate 8.1% of the government budget for the COVID-19 response, greater than the previous limit of 3% that would have otherwise required parliamentary consent to exceed.

Indeed, additional funding also came in many cases through reallocation of resources and leveraging of various regulations related to emergencies. In Ukraine, funds for the COVID-19 health sector response came from different budgetary lines including the special Government Fund to Fight COVID-19 and its Consequences (COVID-19 Fund), the Program of Medical Guarantees (benefit package), Ministry of Health programs and from budget lines of other government agencies (e.g. Ministry of Internal Affairs, National Academy of Science), as well as from local government budgets. In Nigeria, additional funds were made available by the government through extra-budgetary and emergency funds allocation, a review of budgets and the Basic Health Care Provision Fund (BHCPF). This was in addition to the highly significant private sector and philanthropic donations. Similarly, in Ghana, the government provided direct funding from general government revenues to the providers through the Ministry of Health. Likewise, in the Philippines, resources from within the health sector budget were reallocated. The national government provided additional funds to Local Government Units (LGUs) in cities, municipalities and provinces as ‘LGU COVID-19 response grants’ for the provision of basic services including health-related responses and support to frontline health workers. The national government also eased restrictions on the use of funds so that LGUs would have more flexibility in the use of their disaster risk management fund and local development fund.

### Extensions in the benefit package

In most countries, the benefit package was expanded to cover COVID-19 testing and inpatient services through public funds, i.e. in principle, without user charges, but with varying conditions (Table 2). COVID-19 benefits were extended to all citizens irrespective of whether they were covered by the national health insurance schemes or not in Ghana (but only in public facilities), as well as in Armenia and Romania. In Cameroon, COVID-19-related services were free only in public hospitals.

**Table 2.** Coverage of COVID-19-related services through public funding

	Testing	Medications	Hospitalization	Isolation	Tele-consultations	Home-based care
Armenia	√	√	√	√	√	√
Cameroon	√	√	√	√	√	√
Ghana	√	√	Only at public facilities	√	Via mobile apps	Only home visits by staff and patients were given free thermometers
Kenya	Only for civil servants	Only for civil servants	Only for civil servants	√	√	√
Nigeria	Only until March 2021	Only until March 2021	Only until March 2021	√	Primarily at private providers	√ (it has continued as an acceptable and cheaper alternative)
Philippines	√	√	√	√	√ (part of home-based care)	Only from 2021
Romania	√	√	√	√	√	√
Ukraine	√	For inpatient care	√	No	√	√

In three countries, the core COVID-19 health services could not be provided free of charge to all citizens. For instance, in Kenya, the benefit package was not expanded for the whole population. Initially, supplies for COVID-19 services were procured by the Kenyan government. However, due to shortages in supply (such as medicines, oxygen or masks), providers started charging for these services. From May 2021, Kenya’s National Health Insurance Fund started charging members for COVID-19 hospitalizations, while only civil servants who were enrolled through a managed scheme—supplemented with additional funding from the Ministry of Public Service—remained covered.

However, despite the provision of free COVID-19 services in most countries, many patients incurred high out-of-pocket expenses, especially from private providers. In Ghana, COVID-19 services provided through the Ministry of Health were in principle free but severely ill patients were asked to pay for expensive drugs and oxygen. In the Philippines, although PhilHealth implemented a no-balance billing policy for its COVID-19 benefits, there were reports that this was not adequately implemented, resulting in out-of-pocket payments for many care seekers. In Nigeria, COVID-19 hospital treatment was considered financially unsustainable in government health services; later in the pandemic, patients were asked to pay for diagnostic and treatment costs, and in March 2021, the benefits were rolled back to the pre-pandemic scope.

Other types of services with new service delivery modes were also introduced in several countries. In the Philippines, Cameroon and Nigeria, costs in isolation centres were covered by the government. However, in the Philippines for instance, due to delays in contracting providers of isolation services at the community level, availability of such services remained low. In Armenia and Romania, the state-funded benefit packages included teleconsultations for home monitoring of COVID-19 (including text messages and a video application) as well as non-COVID-19 care. The uptake of these services was high and helped to sustain utilization despite COVID-19 mobility restrictions. Other countries also adopted mobile health solutions to varying degrees. In Ukraine, health facilities were allocated additional funds from government to set up mobile teams for COVID-19 sample collection, an advance that was later integrated into primary health care. Teleconsultation was also provided in the Philippines and Nigeria. For instance, in the latter, teleconsultations were primarily in the private sector and mostly limited to phone calls to report suspected cases and for providing follow-up services for home treatment and self-isolation.

Further, despite the benefit package adjustments and extensions in all countries, there were also reports of poor quality of care because of shortages in medical supplies, mainly PPE and oxygen. To some extent, this was due to the disruption in global supply chains.

### Adjustments in provider payments

Most countries in this study adjusted their provider payment methods and processes in response to the pandemic (Table 3). This served to compensate for additional costs or to strengthen incentives to provide COVID-19 health services. While provider payments are decisive to ensure that funds are translated into the promised COVID-19-related health benefits, country experiences suggest that payment adjustments were very challenging. The Philippines is an example of a country which adopted multiple changes and adaptations to its payment methods through the pandemic. The low tariffs set under the initial case-based payments for COVID-19 services led to a backlash from providers. The return to case-based payments again later resulted in cost containment issues for PhilHealth despite the increase in COVID-19 cases since the average payment per claim for COVID-19 admissions was reduced. Also, PhilHealth reactivated a special advance payment model that had already been used during a previous natural disaster, which consisted of frontloading 3 months’ worth of claims payments based on historical data to hospitals, maternity care providers and freestanding dialysis clinics to ensure the continuous provision of these services. However, the policy was subjected to a legislative inquiry and its implementation was eventually suspended in 2020 as it was potentially prone to fraud.

**Table 3.** Adjustments in provider payments

	Adjustments
**Armenia**	Initially, providers were reimbursed based on the full cost of COVID-19 treatment, but later case-based payments were applied
**Cameroon**	Payments are made directly to providers at district level without passing through the centralized and rather long official channel as required by the PFM law. In remote areas, mobile money platforms were used
**Ghana**	Providers were (supposed to be) paid based on fee-for-service, upon having submitted their claims directly to the Ministry of Health (as COVID-19 health services were not covered under the national health insurance scheme)
**Kenya**	The government supplied COVID-19 medical supplies and equipment directly to health facilities
**Nigeria**	No changes applied (fee for service and capitation remained in place)
**Philippines**	PhilHealth used different payment methods: Initially from February 2020 case-based payments for COVID-19 services; from March 2020 onwards fee for service to cover all costs of hospitalizations for only health care workers; and from April 2020 case-based payments, costed according to defined clinical guidelines
**Romania**	Initially, providers were reimbursed based on the full cost of COVID-19 treatment, but later case-based payments were applied
**Ukraine**	Global budget for mobile COVID-19 teams and for hospitals and emergency services (which were adjusted by number of services provided and COVID-19 incidence); the capitation payment rate for primary care services was increased by 8.5% in November 2020

For both purchasers and providers, as seen in the Philippines and elsewhere, it was difficult to cope with the multiple modifications to its provider payment methods to account for the changing cost of delivery of COVID-19 services. Although changes in the provider payment mechanisms attempted to offer greater flexibility to providers, reimburse them for COVID-19 services and incentivize health workers at the frontline, these adaptations were accompanied by various implementation challenges. For instance, in Ukraine, the COVID-19 pandemic coincided with the planned start of the provider payment reform of specialized care; hence, both purchasers and providers had little experience working within the new framework. This created accountability challenges with the use of funds—some providers kept money in their accounts instead of using them for providing COVID-19 services. Evidence from Armenia and Romania suggests that the provider payments were perceived as insufficient, and this has been likely the case in other countries as well. In Ghana, only a small fraction of claims submitted by the providers had been reimbursed by December 2021. This was because the Ministry of Health did not have sufficient funds to cover these costs, and shortfalls caused severe resource constraints for providers, necessitating them to start charging seriously ill patients for costly drugs and oxygen.

In several countries, the government decided to pay salary top-ups to health staff providing COVID-19 services, but this was challenging to implement (Table 4). In Ghana, except for tax relief, staff did not receive other incentives that were promised. Likewise, in Nigeria, the additional incentives provided to staff were delayed and short-lived because of high costs, although this was eventually resolved to avoid industrial disharmony. In Kenya, part of the extra-budgetary transfers from the federal to the county governments was meant to compensate health workers for extra time and to pay allowances and medical coverage for the retired medical staff and medical trainees who volunteered to participate in the response. Unfortunately, these funds were not disbursed, also leading to strikes. A somewhat difficult challenge emerged in Armenia, Romania and Ukraine, where additional incentives to staff providing COVID-19 health services also triggered similar demands for financial incentives from non-COVID-19 service providers. In Ukraine, for instance, this also led to demands by local governments and hospitals to increase the number of designated COVID-19 providers, impacting the budget.

**Table 4.** Compensation for health workers providing COVID-19 services

	Salary top-ups or bonuses	Other benefits
Armenia	√	Bonuses were paid to doctors, nurses and ambulance workers
Cameroon	√	Extension of the retirement age of public health workers from 55 to 60 years
Ghana	√ (base salary increased by 50%)	Life insurance, income tax waivered for 6 months
Kenya	Planned, but not put in practice	-
Nigeria	√	During the nationally declared period of the pandemic: frontline health workers received short-term bonuses while those in isolation and treatment centres received in addition a special transport allowance. Hazard allowance for health workers received an upward review in December 2021, which was implemented in the last quarter of 2022.
Philippines	√ (some top-ups through the national budget such as hazard pay or Special Risk Allowance for public and private providers for both temporary workers and staff)	Full coverage of direct health care costs by PhilHealth for COVID-19 hospitalization during the declared public health emergency; additional monetary compensation to public and private health workers who contracted COVID-19 or died in the line of duty.
Romania	√	Monthly risk payments and per capita incentives for monitoring COVID-19 patients remotely and salary adjustments
Ukraine	√	-

### Modifications in provider selection and contracting

Provider selection and contracting are central purchasing instruments to ensure that providers meet quality standards and establish payment and service delivery terms and conditions. These instruments were equally critical during the pandemic. To better meet the demands of the health systems during the pandemic, all countries in this study modified their contracting modalities and processes of accreditation (i.e. a review process of providers of meeting quality-related standards and requirements). Overall, the type of adjustments applied aimed to ensure quality provision of the full range of COVID-19 services and expand the number of providers by both contracting in and contracting out, while taking account of their existing institutional capacity.

Practically, most purchasers faced a dual task: revisit how they contract providers while rapidly extending services. On the one hand, they needed to re-examine existing accreditation and contracting modalities to incorporate COVID-19-specific requirements. In Ghana, for example, a Presidential Executive Instrument was issued for all public hospitals to treat COVID-19 patients only after going through a training program. As such, the government tightened up the accreditation process to ensure that public providers were able to safely treat COVID-19 patients. In other countries, contracting modalities were reviewed not only to incorporate COVID-19 requirements (e.g. related to clinical practice, case management), but also to introduce some additional incentives to strengthen the service provision capacity of contracted facilities and reward staff dedication. For example, in Ukraine, designated facilities that fulfilled service delivery requirements were contracted and offered readiness packages in April 2020 for COVID-19 inpatient care, including resources to provide salary top-ups. These facilities also received additional investments, e.g. for oxygen supply, equipment and refurbishment needs.

On the other hand, the purchaser had to rapidly extend the number and the types of health facilities and health personnel they could purchase services from, including laboratories, tracking and tracing service providers and isolation centres. In several countries, when the demand for services became overwhelming, accreditation processes were relaxed to expand coverage via private providers. In Armenia and Romania, for example, private medical laboratories were permitted to offer COVID-19 testing based on signed orders. Moreover, in Armenia, as in Ghana, accreditation processes for COVID-19 services were opened to private providers. In Nigeria, both national and state governments were purchasing COVID-19 services from the private sector, and private medical laboratories were accredited to increase COVID-19 testing capacity.

In the Philippines, the urgent need to expand the number of providers also opened a window to simplify the accreditation process. Two key decisions were made: first, health providers who were applying for accreditation under PhilHealth were granted ‘provisional’ accreditation upfront which allowed them to submit claims before being officially accredited. Second, PhilHealth decided to automatically accredit providers which had been licenced by the Department of Health, therefore aligning its accreditation requirements with those of the licencing process. As such, the crisis led to greater synergies with the Department of Health, since, before the pandemic, these two processes were run in parallel and separately, with inevitable operational overlaps and inefficiencies.

### Making use of information management systems

The availability of accurate and real-time information is crucial for the national health response to COVID-19 and specific purchasing decisions. Governments have two key tasks in this area: tracking the epidemiological trends and monitoring the use of COVID-19 and other essential services—i.e. who provided what services, how, when and to whom. Measures taken in the information management system varied across countries, with the pre-existing level of data integration as a key determinant. While all countries managed to set up a COVID-19 monitoring system, those which had reached some level of data integration across purchasers prior to the crisis were better able to track the use of COVID-19 services.

In some countries, the journey towards integration of the different data streams in the health sector had already been initiated before the crisis through the establishment of national unified data platforms on service use. In such contexts, these platforms were used to fulfil both the functions of surveillance and monitoring service use. The crisis has revealed the benefits of such unified platforms. In Armenia, the national digital health system *ArMed*, established in 2017, enabled the collection and integration of clinical, administrative and financial data on the provision of standard health services. With the addition of an integrated COVID-19 module, the Ministry of Health could access a wealth of seamlessly transmitted data and take informed decisions related to purchasing as well as disease control measures. For the Ministry of Health, the pandemic was an eye-opener on the importance of such integrated data platforms, and plans to further expand the scope of captured data are already being discussed. Likewise, in Ukraine, the government used the pandemic to speed up the upgrade of such a unified data platforms that included data on hospital COVID-19 cases.

In the Philippines, there was partial integration of data: the PhilHealth information system collected data on COVID-19 treatment and financial costs. However, because of limited integration with other data streams, it was inadequate to steer the national response. Due to delays in claim submission, it was also difficult to generate timely information, and the Department of Health had to maintain a parallel surveillance reporting system for the pandemics. While theoretically, the two systems are complementary, in practice, health providers complained about overlapping reporting requirements.

In countries with limited data integration and where the purchasing mandate was split over multiple institutions, existing data siloes persisted. Information on the two functions (disease surveillance and service use monitoring) was captured by separate data streams. For surveillance, countries like Nigeria, Kenya, Ghana and Cameroon reinstated and updated existing incident management systems used in previous health crises. Because these systems were familiar to users, they could be deployed rapidly. However, the reinstated surveillance data systems were not flawless and faced problems such as limited data range and data quality issues. Moreover, these systems provided limited insights into COVID-19 service use. The data from the purchasers also did not provide these data or the data produced were so heterogenous that it was difficult to consolidate it across multiple purchasers.

## Discussion

Our paper provides a novel perspective and more detailed insights on purchasing adjustments that were made by health systems as a response to COVID-19 in the selected MICs. Unlike low-income countries, these MICs have relatively more resources, both financial and technical, to respond to crises such as the COVID-19 pandemic and all have a national purchasing agency, although the health financing system is still fragmented. We identify several commonalities across countries, notably the adjustments in payment methods, expansion of benefit packages to cover COVID-19-related health services and provision of additional hardship and motivational payments to health workers, whilst experiencing funding challenges to finance these, and in many cases, the need to rely on external financing. However, there were also significant differences, including the ability of countries to use information management systems to drive the response and the way the private sector was leveraged. Overall, these findings are in line with earlier studies on purchasing responses to COVID-19 in LMICs (Haldane *et al*., 2021; Gadsden *et al*., 2022; Mathauer *et al*., 2022; SPARC, 2022). Yet, they are in stark contrast to how the health sector responded in high-income countries, in particular, by adjusting their payment methods, backed by additional funding, although with a closer look, there were also variations found within this country group. One commonality with high-income countries, however, relates to the rather passive role of purchasers, such as under social health insurance schemes, in COVID-19-related decision-making in purchasing (Montás *et al*., 2022; Schmidt *et al*., 2022).

It is difficult to provide a comprehensive explanation of the factors influencing decisions due to the confluence of different variables that were driving decision-making at the time of the pandemic. However, key explanatory factors for these differences may relate to varying degrees of political willingness and leadership by decision-makers, the ability to effectively coordinate a complex response to a novel threat across multiple levels of government and the overall availability of funds.

In a nutshell, this synthesis reveals that alignment between the adjustments in the key domains of purchasing, as outlined in the conceptual framework, and service delivery capacities were crucial to ensure the provision of new COVID-19 services while maintaining the provision of essential non-COVID-19 care. Our comparative analysis across these eight countries outlines the main adjustments found and provides a deeper understanding of the multiple implementation challenges, based on which, we identify several policy implications for strengthening strategic purchasing, as outlined below.

### Importance of flexibility and alignment in purchasing adjustments

Foremost, mobilizing more funds and providing adequate and flexible funding to providers for COVID-19 services were critical, as confirmed elsewhere (Barroy *et al*., 2020; 2022; Wee-Co *et al*., 2022; World Health Organization, 2022). All countries needed to find additional sources of funding and reallocate existing resources to expand their benefit packages in order to provide free testing, hospitalizations and medications for COVID-19, while some also provided free community isolation (Cameroon, Nigeria, Philippines) and teleconsultations for home care (Armenia, Romania, Philippines). These trends are in line with other findings (Mathauer *et al*., 2022; SPARC, 2022). However, as the benefit extension measures were not aligned with available funding in all countries—in some, these turned out to be inadequate, delayed or inflexible—costs ended up being passed on to patients (Moynihan *et al*., 2021; Barron *et al*., 2022).

Access to timely information was essential in making strategic choices. However, some countries had to pause their plans to improve their information management and monitoring systems, while other countries’ leaders recognized this window of opportunity and used the pandemic to accelerate information management development (Ukraine). Countries that had already implemented incident management systems (Cameroon, Ghana and Nigeria) highlighted the benefits of integrated information management systems, suggesting that although the pandemic was a stress on the health system, it also provided an opportunity to advance innovation and support alignment across different purchasing measures.

In all countries, the COVID-19 response was primarily delivered through the public health system, with the private sector bridging the gaps in public provision. This re-emphasizes the calls for supporting an adequately funded and efficient public health system while promoting collaborations and partnerships as well as alignment with the private sector (Baxter and Casady, 2020; Tsilaajav *et al*., 2020; Tan *et al*., 2021). In some countries, this was achieved by simplifying the contracting and accreditation processes for private providers to support an aligned response.

### A central role for governance and leadership

Results also highlight the importance of establishing robust governance structures for accelerated decision-making, along with the setting of clear mandates and reporting standards, which is in line with other studies (Mathauer *et al*., 2022). However, the lack of clear guidelines and tensions between the purchasers and service providers could lead to uncertainty and mistrust (Jennings *et al*., 2021), as observed in Kenya when delays in payments resulted in strikes.

In most countries, the national COVID-19 response was supported by political leadership at the highest level. This was critical to support policy actions such as commitment to continued funding and multi-sectoral collaborations; yet, funding constraints made it harder to maintain benefit package extensions in LMICs. While decision-making was decentralized in some countries, it is notable that there is overall little or no involvement of wider stakeholders, especially civil society organizations. In some countries, central health sector actors such as health insurance agencies were not included in decision-making. This may be explained by the way health policies are generally designed in these countries, the role that civil society plays during health emergencies, by the specific political economy challenges within each country between agencies or by the regulatory or emergency response role being delegated to a particular agency.

### Uncertainty regarding the sustainability of changes introduced during COVID-19 and the effects of these changes on universal health coverage objectives

The extent to which either beneficial or non-beneficial changes made in purchasing arrangements are retained and whether these changes have any long-term effects on UHC is uncertain. For instance, to ensure financial protection, a few countries rolled out zero co-pay policies for COVID-19 care packages in public health facilities (such as Nigeria) or made advance payments to facilities (such as the Philippines), so as to enable equitable access to health services, but needed to cancel these due to financial sustainability concerns.

However, there is some indication that a few countries may continue or even scale up the advancements made in purchasing arrangements and have positive effects on UHC. For instance, teleconsultations, which contribute to making access more equitable, will be continued in Armenia and Romania following the pandemic. In Armenia, telephone consultations were used more and more for minor COVID-19 illnesses and other services, while in Romania, they were added as a reimbursable service to the Framework Contract that governs provider contracting and payment (Haldane *et al*., 2021). Moreover, payment mechanisms that allow Romanian providers wider flexibility and spending autonomy were piloted during the pandemic and it has been argued to maintain this to increase efficiency, whilst also ensuring enhanced accountability. In these countries, the pandemic may have been used as a jumping board to accelerate needed reforms.

Another important question is to explore the effects of the adjustments made in the purchasing arrangements and whether they support strategic purchasing and ultimately UHC. While changes to purchasing arrangements were made as a part of the COVID-19 health system response, not all the changes might be deemed as strategic purchasing that would positively contribute to UHC. In this study, the term purchasing and not ‘strategic purchasing’ has been employed intentionally given that these changes in purchasing arrangements cannot yet be easily assessed in terms of their outcomes, more so in view of other contributing factors. What is important for purchasing to become more strategic is the degree to which the various purchasing policy instruments help to contribute to UHC objectives of efficiency, equity, financial protection or quality of services along a continuum and over time. Evidence on the effectiveness of purchasing adjustments was limited at the time of the study and overall mixed, in particular in relation to financial protection and equitable access to health services. Nonetheless, in some cases, the strategic nature of the adjustments is evident and there are indications of positive effects related to UHC objectives. For example, there were advances in relation to the provision of telehealth services and the integration of the private sector, as a way to increase equitable access to health services. As other findings suggest, such purchasing adjustments can provide room for innovation by harnessing the private sector, as well as adapting to changing needs that can lead to increased efficiency, digitalization and patient responsiveness, thus also supporting quality objectives (Mathauer *et al*., 2022).

However, implementation challenges coupled with a lack of funding also meant that UHC objectives were not consequently pursued in some countries. When providers did not receive timely and adequate supplies, they passed on the costs to patients, putting patients at risk of financial catastrophes when affected by COVID-19. The largest concern relating to UHC objectives was hence the negative effect on financial protection, particularly for the poor. Or else, there was the unintended consequence of neglect or non-availability of other essential health services, because health workers were either overwhelmed or these services were not adequately funded, thus endangering equitable access to health services as well as quality care (Blanchet *et al*., 2020). Moreover, there also seemed to be challenges around multiple revisions of purchasing policies and clear guidelines/communication to providers and/or subnational units, which negatively affects efficiency and transparency.

### Limitations

Using an open platform to solicit participation from country teams meant the eight country studies do not evenly represent MICs and rather represent the interest of participants to share and participate in cross-country learning. There was no country from the Eastern Mediterranean region, South America or South Asia. Moreover, the depth of evidence from the eight countries varied depending on the time and resources available to the local teams. Since the data were collected during the pandemic, it was difficult to conduct interviews. Comparison was further limited because of different methodologies used across the country studies and limited data collected on contextual factors that may have influenced the health systems response. Further, there were limited quantitative data available on financial protection, quality of care and equitable access; thus, we could not firmly conclude whether these purchasing adjustments contributed to progress in UHC objectives during COVID-19.

Finally, based on the data collected from March to November 2021, we could not examine the impact of purchasing adjustments or assess how they might have further evolved following the pandemic. These findings represent a specific timeframe in the pandemic and countries’ purchasing adjustments may have further evolved.

## Conclusion

Our synthesis illuminates the crucial role of the set of adjustments made in purchasing arrangements in supporting the COVID-19 health sector response in all countries. While some countries faced significant implementation challenges and alignment gaps, in other countries, it was possible to use these changes as stepping stones to more enduring changes in purchasing arrangements. Few changes had been pursued incrementally, and many were rather abrupt.

There were challenges to move from policy changes to implementation due to capacity and timing constraints. Insufficient clarity in guidelines and processes can help to explain some of the gaps between design and implementation. There were also tensions between the mandate assigned to a purchaser and the funding given to undertake that mandate. As such, the lack of funding and implementation challenges likely limited the envisaged objectives of these adjustments in purchasing arrangements, with a major concern relating to inadequate benefits as well as increased out-of-pocket expenditure for COVID-19 services due to insufficient resources and untimely payment of providers. Ultimately, it will be important to have pre-established governance arrangements that allow for decision-making in emergencies, where it is critical to involve a broader set of stakeholders, such as health insurance funds, NGOs and civil society organizations. Countries should also have an understanding of how new or existing resources may be mobilized towards emergencies as needs become apparent.

For purchasing arrangements to be a strong support to service reconfigurations when a health emergency occurs, our findings also suggest that purchasing arrangements need to have sufficient built-in adaptability, as well as adequate information systems to assess whether changes are meeting needs as a health emergency progresses and requirements evolve. In that sense, strategic purchasing is also a relevant tool for improving resilience and therefore instrumental to pandemic preparedness.

## Supplementary Material

czad121_Supp

## Reflexivity statement

The authors include 13 females and 11 male and span multiple levels of seniority. Six authors are based in Africa, six authors work in Eastern Europe, four authors each are located in the USA and in Western Europe, respectively, while three authors are based in the Philippines, and one author is based in Colombia. Seven authors are based at universities or research institutes, 10 work for international organizations, whereas two are with ministries of health and five are with international or national NGOs. While all authors specialize in health systems strengthening and health financing including purchasing in LMIC, eight have specific expertise in strategic purchasing globally. All authors have experience conducting fieldwork and data analysis.
