# Supplementary material for: Adjustments in purchasing arrangements to support the COVID-19 health sector response: evidence from eight middle-income countries
Source: Health Policy Plan. 2024 Feb 9;39(2):213–23. doi: 10.1093/heapol/czad121 (PMC10883665; doi:10.1093/heapol/czad121)
Supplement: czad121_Supp [file czad121_supp.zip › Supplementary file_11102023_final.docx]

**SUPPLEMENTARY FILE**

**Table S1: Key information on countries included in the study**

| **Country** | **GDP per capita (USD)^a^** | **Poverty below 2.15 USD/day, 2017 PPP (%)^a^** | **Life expectancy (years)^a^** | **Population^a^** | **Income group in 2021^a^** | **Out-of-pocket expenditure as percentage of current health expenditure, 2019^b^** | **UHC index - Service coverage, 2019^c^** | **UHC coverage index - Financial protection^d^** | **Cumulative number of confirmed COVID 19 cases as of 31 December, 2021** |
| --- | --- | --- | --- | --- | --- | --- | --- | --- | --- |
| Armenia | 4,670 | 0.4% | 75 | 2,968,128 | Upper middle | 84.80% | 69 | 20.98% (2017) | 344,826 |
| Cameroon | 1,662 | 25.7% | 60 | 27,224,262 | Lower middle | 72.50% | 44 | 10.71% (2014) | 109,367 |
| Ghana | 2,445 | 25.3% | 64 | 31,732,128 | Lower middle | 36.20% | 45 | 1.33% (2016) | 142,986 |
| Kenya | 2,007 | 29.4% | 67 | 54,985,702 | Lower middle | 24.30% | 56 | 5.05% (2015) | 292,237 |
| Nigeria | 2,085 | 30.9% | 55 | 211,400,704 | Lower middle | 70.50% | 44 | 15.79% (2018) | 241,513 |
| Philippines | 3,549 | 3.0% | 71 | 111,046,910 | Lower middle | 48.60% | 55 | 6.31% (2015) | 2,841,260 |
| Romania | 14,862 | 2.2% | 74 | 19,115,146 | Upper middle | 18.90% | 71 | 13.42% (2016) | 1,807,223 |
| Ukraine | 4,836 | 0.0% | 71 | 43,814,581 | Lower middle | 43.90% | 73 | 8.32% (2019) | 3,840,041 |
| ^a^ Source: World Bank <https://data.worldbank.org/country> (assessed on 5 October 2022)  ^b^ Source: WHO <https://apps.who.int/nha/database/country_profile/Index/en> (assessed on 5 October 2022)  ^c^ Source: WHO <https://www.who.int/data/gho/data/major-themes/universal-health-coverage-major> (assessed on 5 October 2022)  ^d^ Source: WHO <https://apps.who.int/gho/data/node.main.UHCFINANCIALPROTECTION01?lang=en> (assessed on 5 October 2022)  ^e^ Source: Our World Data <https://ourworldindata.org/coronavirus> (assessed on 5 October 2022)  Abbreviations: GDP Gross Domestic Product, PPP Purchasing Power Parity; UHC Universal Health Coverage | | | | | | | | | |

**Table S2. Overview of key purchasing agencies**

|  | **Main purchasing agencies** |
| --- | --- |
| **Armenia** | State Purchasing Agency; Ministry of Health; 6 private insurers (administering the publicly funded package for government employees) |
| **Cameroon** | Ministry of Public Health, The Regional Fund for Health Promotion (purchaser for many schemes such as the PBF and the Health Voucher), National Social Insurance Fund Scheme |
| **Ghana** | Ghana Health Services, National Health Insurance Agency (both separate purchasers) |
| **Kenya** | Ministry of Health; National Health Insurance Agency (separate purchaser) |
| **Nigeria** | Federal and State Ministries of Health; National and State Health Insurance Scheme purchased only other essential healthcare services |
| **Philippines** | Department of Health, PhilHealth (national health insurance scheme) |
| **Romania** | National Health Insurance House, District Health Insurance Houses, Ministry of Health |
| **Ukraine** | National Health Service of Ukraine (national agency financed from general taxation and responsible for strategic purchasing of guaranteed health services) |
| Source: Authors | |

**Table S3: Data collection methods of the country studies and sources used for the synthesis paper**

| **Interviews and surveys** | | **Documents reviewed for the country studies** | | **Secondary data used for the country studies** | | **Source(s) used for this synthesis paper** | |
| --- | --- | --- | --- | --- | --- | --- | --- |
| **Armenia** |  | |  | |  | |  |
| *Interviews*  N = 10  (4 male, 6 female)  2 Ministry of Health; 1 State Health Agency; 3 Health service users; 4 Health providers. | | - European Observatory on Health Systems and Policies. 2021. "COVID-19 Health System Response Monitor (HSRM) Armenia." Brussels, Belgium. <https://eurohealthobserva-tory.who.int/monitors/hsrm/hsrm-countries/hsrm/armenia> . - The Health Component of the Social Package: A Cross-Sectional Sur-vey of Beneficiaries." American University of Armenia, Yerevan - <https://ncdc.am/coronavirus/confirmed-cases-by-days/>, Data consulted as of July 31, 2021. - WHO Country Office Team, Armenia. (2020). Covid-19 Health System Response Monitor: Armenia.https://www.covid19healthsystem.org/countries/armenia/countrypage.aspx - An actuarial model for costing universal health; 2020 Technical support towards universal health coverage in Armenia. | | - Database on confirmed COVID-19 cases <https://ncdc.am/coronavirus/confirmed-cases-by-days/> - Health and Healthcare, Statistical Yearbook, 2020. <http://nih.am/am/statistical_yearbooks/120/am> | | Chukwuma A, Wang H, Chen D, Gong E, Rivillas J. COVID-19’s Impact on Strategic Health Purchasing: Lessons from Armenia and Romania.2021; [accessed 2022 Nov 14]. <https://r4d.org/blog/covid-19s-impact-on-strategic-health-purchasing-lessons-from-armenia-and-romania/>.  Chukwuma A, Rivillas J, Gong E, Wang.H, Chen D, Lylozian H, Comsa R, Pirjol D, Dmytraczenko T. Purchasing in a pandemic: Lessons from the health systems response in Armenia and Romania. 2023; USA: Europe and Central Asia Region. Health, Nutrition, and Population Global Practice, World Bank Group. | |
| **Cameroon** | | | | | | | |
| *Interviews*  N=6  (Respondent data on sex not reported)  1 Medical advisor; 1 District medical officer; 2 Performance-based financing administrators, 2 Health providers. | | - COVID-19 Brief circulars from Ministry of Health - Government procedures related to COVID-19 purchasing. - Government response strategy to the coronavirus pandemic (COVID-19 <https://www.prc.cm/en/multimedia/documents/8228-government-response-strategy-to-the-coronavirus-pandemic-covid-19> - Ministry of Health reports <https://minepat.gov.cm/?s=covid> - Human Rights Watch 2020 <https://www.hrw.org/node/376484/printable/print> | | - [WHO Coronavirus Disease (COVID-19) Dashboard](https://covid19.who.int/region/afro/country/cm) | | Nkangu M, Gobina M, Nkengafac F, Maabo J, S F. COVID-19’s Impact on Strategic Health Purchasing: Lessons from Cameroon.2021; [accessed 2022 Nov 14]. <https://r4d.org/blog/covid-19s-impact-on-strategic-health-purchasing-lessons-from-cameroon/>. | |
| **Ghana** | | | | | | | |
| *Interviews*  N=9  (7 male, 2 female)  3 Ministry of Health; 2 Ghana Health Service; 1 National Health Insurance Authority; 2 Health providers; 1 Ghana Infectious Disease Centre. | | - Ghana Health Sector 2021 Programme of Work - Ghana COVID-19 Emergency Preparedness and Response Project & Additional Financing: Environmental and Social Management Framework (ESMF). - National Strategic COVID-19 Response Plan: July 2020 - December 2024Ministry of Health. (2020). - Republic of Ghana Ministry of Health. (2020). Provisional Standard Treatment Guidelines for Novel Coronavirus Infection COVID-19 Guidelines for Ghana. - National Guidelines for Laboratory Testing and Reporting on Respiratory Infectious Diseases Ghana | | - COVID-19 Ghana's Outbreak Response Management Updates (2020-2021)   <https://ghs.gov.gh/covid19/> | | Adin-Darko D, Duker A, Fenny A, Bloom D. How COVID-19 affected vaccine procurement processes in Ghana.2021; [accessed 2022 Nov 14]. <https://r4d.org/blog/how-covid-19-affected-vaccine-procurement-processes-in-ghana/>.  Abuosi A, Nketiah-Amponsah E. Adjustments in Purchasing Arrangements as Part of the Health Sector Response to Covid-19 in Ghana. Final WHO Consultancy Report, Ghana. 2022 (unpublished); Ghana: World Health Organization Country Office. | |
| **Kenya** | | | | | | | |
| *Interviews*  N = 2  (2 male)  1 National Health Insurance; 1 Key informant | | - Updates on COVID-19 from the Ministry of Health’s National Emergency Response Committee on Coronavirus: <https://www.health.go.ke/press-releases/> - COVID-19 news and press releases from April 2020- November 2021 - Blogs and commentaries from July 2020 (Development Initiatives) - Health Budget 2021-2022 - National budget statement 2020/21 - National Health Insurance Reports 2020-21 - Kenya National Gazette and Parliamentary Sessions 2020 - Supplier analysis of COVID-19 contracts awarded by the county and national Governments in Kenya. <https://tikenya.org/wp-content/uploads/2021/05/SUPPLIER-ANALYSIS-OF-COVID-19-CONTRACTS-AWARDED-BY-THE-COUNTY-AND-NATIONAL-GOVERNMENTS-IN-KENYA_2.pdf> | |  | | Njoka J, Oneko T, Tapkigen J. Adjustments in purchasing arrangements as part of the COVID-19 health sector response in Kenya, Collectivity Project, unpublished report, 2022; Nairobi. | |
| **Nigeria** | | | | | | | |
| *Interviews*  N= 8  (Respondent data on sex not reported)  3 National COVID-19 Isolation Centres; 2 Health Maintenance Organizations; 1 Federal Ministry of Health; 1 Federal Ministry of Finance; 1 National Health Insurance Scheme (NHIS). | | - NHIS Operational Guidelines - Nigeria Health Budgets 2020 and 2021 - Nigeria Health Financing Policy and Strategy 2017 - National Strategic Health Development Plan II (2018-2022). | |  | | Ayomoh F, Obikeze O, Onwujekwe O, Orji N, Peters E. Strategic health purchasing for the COVID-19 pandemic response for building back better: A case study of Nigeria. Collectivity Project, unpublished report, 2022; Abuja. | |
| **Philippines** | | | | | | | |
| *Interviews*  N = 6  (2 male, 4 female)  2 PhilHealth; 1 Private HMO; 2 Health providers; 1 patient representative. | | - Directorate of Health (DOH) policies - PhilHealth circulars on COVID-19 - COVID-19 related legislations - News and media coverage | | - PhilHealth COVID-19 Claims data - DOH COVID-19 data <https://doh.gov.ph/2019-nCoV> | | Wee-Co P, Claro V, Domingo A, Pattnaik A, Vilcu I, Dkhimi F. COVID-19’s Impact on Strategic Health Purchasing: Lessons from the Philippines. 2021; Online: Results for Development. | |
| **Romania** | | | | | | | |
| *Interviews*  N = 14  (6 male, 8 female)  1 Ministry of Health; 1 Ministry of Finance; 2 National Health Insurance; 1 National Health Insurance Housel  5 Health service users; 5 Health providers. | | - Institutul National de Sanatate Publica (INSP) Covid-19 Report - National Institute of Statistics, Demographic statistics, Romania, Data consulted as of July 31, 2021 - Centrul Național de Supraveghere şi Control al Bolilor Transmisibile - Analiză date supraveghere (cnscbt.ro) - State of Health Report 2019 <https://insp.gov.ro/wpfb-file/raport-starea-de-sanatate-2019-pdf/> - European Observatory on Health Systems and Policies. Adjusting hospital inpatient payment systems for COVID-19 - Policy responses for Romania. Health financing measuring introduced in 2020. | | - National Institute of Statistics, Demographic statistics - Data on COVID infections and vaccination <https://data.gov.ro/dataset/transparenta-covid> - <https://ec.europa.eu/eurostat/databrowser> - Coronavirus (COVID-19) Cases reported in Statistics and Research Our World in Data, | | Chukwuma A, Wang H, Chen D, Gong E, Rivillas J. COVID-19’s Impact on Strategic Health Purchasing: Lessons from Armenia and Romania.2021; [accessed 2022 Nov 14]. <https://r4d.org/blog/covid-19s-impact-on-strategic-health-purchasing-lessons-from-armenia-and-romania/>.  Chukwuma A, Rivillas J, Gong E, Wang.H, Chen D, Lylozian H, Comsa R, Pirjol D, Dmytraczenko T. Purchasing in a pandemic: Lessons from the health systems response in Armenia and Romania.2023; USA: Europe and Central Asia Region. Health, Nutrition, and Population Global Practice, World Bank Group. | |
| **Ukraine** | | | | | | | |
| *Interviews*  N = 10  (6 male, 4 female)  2 Regional health administrations; 8 Health providers  *Telephone survey*  N = 2121  (914 male, 1207 female)  representative sample  Patients (adults 18+ who confirmed having a COVID-19, diagnosed by COVID-test) | | - Government budget - Law of Ukraine on Program of Medical Guarantee - Cabinet of Ministers regulations on payments - Ministry of Finance reports - Mistry of Health, Center of Public Health, National Health Service of Ukraine (NHSU) reports on COVID-19 situation - COVID-19 response plans | | - Claims data 2021 - Ministry of Health data on COVID-19, statistics on COVID-19 tests and COVID-19 vaccinations | | Doroshenko O, Goroshko A, Pak K, Shekar V, Khan O, Bredenkamp C. COVID-19’s Impact on Strategic Health Purchasing: Lessons from Ukraine.2021; [accessed 2022 Nov 14]. <https://r4d.org/blog/covid-19s-impact-on-strategic-health-purchasing-lessons-from-ukraine/>. | |

**Table S4: Definition of purchasing domains under study and related thematic areas guiding data analysis**

| **Purchasing domain** *(definition)* | **Thematic areas for data analysis** |
| --- | --- |
|  |  |
| **Governance arrangements**  *(An overarching health systems function for ensuring that strategic policy frameworks exist and are combined with effective over-sight, coordination, coalition-building, regulation, attention to system-design and accountability)* | - Modifications in existing structures for decision-making or policy discourse - Establishment of new governance structures to coordinate the overall national COVID-19 response - Reliance on centralized governance arrangements versus making use of subnational government arrangements - Identification of lead ministry or agency in the COVID-19 (health sector) response - Composition of COVID-19 decision-making bodies and level of stakeholder engagement - Role of the national health insurance schemes (in those countries where present) - Challenges in policy design and implementation |
| Mobilizing and reallocating funds(Increasing revenue for health from new sources or reallocating funds from existing health or non-health priorities) | - Commitment of additional donor financing - Deployment of emergency financing - Reallocation from overall government budget - Reallocation from within the health sector budget - Creation of special fund holding arrangements to manage resources - Legal changes to allow for flexibility in expenditure - Use of existing channels to funnel resources to COVID-19 priorities - Identification of new private sector resources - Challenges in policy design and implementation |
| **Benefit package** (The set of health services, including drugs, that are covered by prepaid and pooled funds) | - Identifying population groups covered by the expanded benefits - Expanding benefits that were included in the package (e.g., COVID-19 testing, medication, hospitalization, isolation, teleconsultations, home-based care) - Changing cost-sharing policies - Challenges in policy design and implementation |
| **Provider payment methods**  *(Mechanisms used to transfer funds from the purchaser to the health providers for provision of health services)* | - Replacement of one provider payment with another - Adding other complementary payment methods - Increasing payment rates - Changing payment transfer processes (mobile money) - Revising level of reimbursement of costs (full versus partial) - Contracting of private facilities - Adjusting level of flexibility given to facilities in relation to the (revised or added) payment methods - Changing user charging practices - Challenges in policy design and implementation |
| **Provider selection and contracting**  *(Identifying service providers ― public, private, and not-for-profit, and the type of contracting mechanisms that will be employed)* | - Including additional providers or new types of providers to deliver services - Changing accreditation processes and criteria - Changing contracting modalities - Adjusting the types of contracting applied (contracting in, contracting out) - Expanding the number of providers for COVID-19 services - Challenges in policy design and implementation |
| **Information management systems**  *(Concerns what information to generate, and how to best manage, analyze and use it for strategic purchasing decisions)* | - Changing the types of data being tracked and monitored - Changing the degree of data integration - Using collected data for purchasing related decision-making - Leveraging innovation and technology for both patient and provider level data collection and back-end data integration and management - Assessing consequences of COVID-19 related data management and monitoring for the broader health information management system - Challenges in policy design and implementation |

Source: Based on Mathauer et al. (2017), Cashin and Gatome-Munyua (2022), and Mathauer et al. (2021)
